# T-Cell Lymphoblastic Leukemia/Lymphoma: Relapse 16 Years after First Remission

**DOI:** 10.1155/2014/359158

**Published:** 2014-04-14

**Authors:** Lauren Elreda, Manpreet Sandhu, Xinlai Sun, Wondwessen Bekele, Alice J. Cohen, Maya Shah

**Affiliations:** ^1^Department of Medicine, Hematology/Oncology, Newark Beth Israel Medical Center, Saint Barnabas Health Care System, Newark, NJ 07112, USA; ^2^Department of Laboratory Medicine and Pathology, Newark Beth Israel Medical Center, Saint Barnabas Health Care System, Newark, NJ 07112, USA; ^3^Department of Pediatrics, Hematology/Oncology, Newark Beth Israel Medical Center, Saint Barnabas Health Care System, Newark, NJ 07112, USA

## Abstract

Little information is available regarding late relapse in patients with T-lymphoblastic leukemia/lymphoma (T-LBL). Because of the aggressive nature of this disease, relapse is common and often happens early. Late relapses are rare and generally occur within a few years after initial remission. The relapse rate after 3 years has been reported to steadily decrease over time yet does not parallel with cure. We report a case of a 26-year-old female with T-LBL and relapse 16 years after her first remission with successful treatment with HyperCVAD and L-asparaginase.

## 1. Introduction

Lymphoblastic leukemia/lymphoma (LBL) is an aggressive subset of non-Hodgkin's lymphoma (NHL) that is derived from B or T-lymphoid progenitors [1]. As a disease comparable to acute lymphoid leukemia (ALL), it represents 2%–4% of all adult NHL and the second most common subtype of NHL in children [2, 3]. With progress in chemotherapy in the past few decades, LBL/ALL has become a curable disease. However, there is still a population of patients who experience relapse and it frequently occurs within the first 2 years or during therapy. Late relapses of T-LBL are rare and have been reported to occur within 3–5 years after remission. In this report, we present a case of a young woman with T-LBL that has recurred 16 years after her first remission and is now successfully treated with HyperCVAD and L-asparaginase.

## 2. Case Report

**Figure 1:**
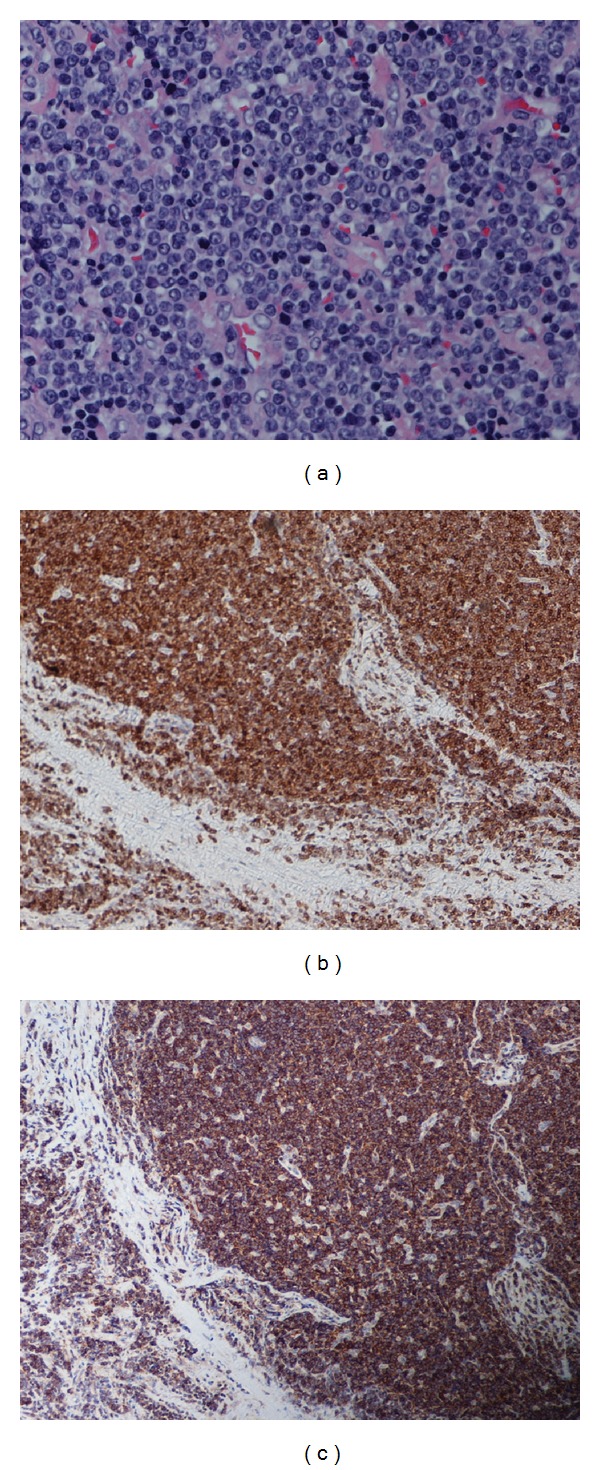
Supraclavicular lymph node biopsy ((a) H&E, 40x; (b) IHC stain for CD3, 10x; (c) IHC stain for CD99, 10x).

A 26-year-old female presented with complaints of right neck swelling for two weeks. She denied any fever, weight loss, or night sweats. On physical examination, right cervical and supraclavicular adenopathy had been present. Biopsy of a supraclavicular lymph node revealed complete loss of lymph node architecture and sheets of monotonous lymphoid cells with scanty cytoplasm that were positive for CD2, CD3, CD5, CD7, CD99, and TdT and negative for LCA by immunohistochemical stain (Figures 1(a), 1(b), and 1(c)) and 40% of cells demonstrated Ki-67 positivity. Focal necrosis was present. Flow cytometry and cytogenetic analysis of the lymph node biopsy were nondiagnostic due to the low viability of the cells. Bone marrow (BM) examination demonstrated focal involvement of precursor T-cells. BM flow cytometry detected 1.5% of T-cell lymphoblasts with similar immunophenotype of cells in the lymph node biopsy. Cytogenetic analysis of the BM was normal. These findings were consistent with T-LBL. Further staging workup included a lumbar puncture which was negative for malignant cells and a PET/CT which revealed metabolic activity in both cervical and thoracic lymph nodes, as well as bilateral inguinal lymph nodes. The clinical stage was Stage IV T-LBL based on the Ann Arbor classification.

Her past medical history is significant for Stage IV T-LBL, involving a right axillary lymph node and bilateral BM involvement, diagnosed at the age of 9. The lymph node biopsy showed malignant lymphoma, lymphoblastic (high grade). The immunophenotype was positive for CD2, CD3, CD5, CD7, TdT, and HLA-DR. The bilateral BM biopsy showed 55% lymphoblasts and 30% lymphoblasts in the right and left sided marrows, respectively. She was treated for a total of 18 months with the LSA2-L2 protocol [4]. This includes a 21-day induction phase with cyclophosphamide 1200 mg/m^2^ intravenously (IV) × 1, daunomycin 60 mg/m^2^ IV × 1, vincristine 2.25 mg/m^2^ IV × 3, prednisone 60 mg/m^2^ orally (PO) × 14 days, and intrathecal (IT) methotrexate × 3. It is followed by a consolidation phase with cytarabine 150 mg/m^2^ IV × 14 days, thioguanine 75 mg/m^2^ IV × 14 days, L-asparaginase 6000 IU/m^2^ IV × 11 days, carmustine 60 mg/m^2^ IV × 1, and IT methotrexate × 2 doses. Maintenance phase includes treatment with thioguanine, hydroxyurea, daunorubicin, both PO and IT methotrexate, and vincristine [4]. No radiation was administered.

At the time of relapse, the patient was not in long-term follow-up.

The treatment was initiated with the HyperCVAD protocol [2]. This includes 8 cycles of alternating chemotherapy. Odd cycles 1, 3, 5, and 7 are comprised of cyclophosphamide 300 mg/m^2^ IV every 12 hours × 3 days with concurrent Mesna, vincristine 2 mg IV on days 4 and 11, doxorubicin 50 mg/m^2^ IV on day 4, and dexamethasone 40 mg PO days 1–4 and 11–14. Even cycles 2, 4, 6, and 8 are comprised of methotrexate 200 mg/m^2^ IV over 2 hours followed by methotrexate 800 mg/m^2^ IV over 22 hours on day 1. This is followed by a leucovorin rescue, dose dependent on the methotrexate levels, and cytarabine 3 g/m^2^ IV every 12 hours on days 2 and 3. IT methotrexate 12 mg was given on day 2 of every cycle and IT cytarabine 100 mg was given on day 8 of every cycle [2]. L-asparaginase 2500 mg/m^2^ IV was added to this regimen and given as the pegylated form with every cycle starting in the second cycle of HyperCVAD. Treatment was tolerated well with little to no adverse side effects. A follow-up bone marrow biopsy and PET/CT scan after treatment revealed no evidence of lymphoma. Maintenance therapy was initiated with POMP (6-mercaptopurine, methotrexate, vincristine, and prednisone) [4] and continued for 2 years.

## 3. Discussion

Precursor lymphoid neoplasms include B- and T-lymphoblastic leukemia/lymphoma. The distinction between leukemia and lymphoma is arbitrary when a patient presents with a mass lesion and lymphoblasts in the BM. LBL is described to be a disease comparable to ALL and often difficult to distinguish from one another morphologically. The major distinction between LBL and ALL is that the degree of BM involvement is often greater in ALL [5]. Most precursor lymphoid neoplasms are B-cell phenotype, but 80–95% of LBL cases are of T-cell phenotype [2]. Our patient initially presented with axillary lymphadenopathy and the BM showed bilateral marrow involvement. Relapse occurred in a cervical lymph node and the BM involvement was minimal. Therefore a diagnosis of T-LBL was rendered.

It has been reported that 20–25% of ALL/LBL patients experience relapse [6]. Relapse in these two diseases is often early and reflects an aggressive disease. In ALL, early relapse is often defined as occurring in the first 1.5 years from diagnosis whereas late relapse is defined as occurring after 3 years from diagnosis and these patients may represent a subset of patients with a distinct biology that follows a more quiescent course [3, 7]. In one long-term follow-up study of 505 children with relapsed ALL, 74% of relapses were observed within the first 3 years and 4% after 5 years. Only 1% of cases relapsed after 10 years and none of them were T-ALL [6]. Similarly, early relapse is much more common in T-LBL than B-LBL [6–8]. It is difficult to ascertain the overall rates of relapse in T-LBL, in particular late relapse, because of the limited data on this subset of patients.

Biologic predictors of relapse have not been well characterized in LBL yet in ALL; early relapse has been recently shown to be a consequence of a distinctive gene over expression which leads to proliferation and survival of the resistant leukemia cell [8]. Also, epigenetic phenomenon such as the presence of TEL-AML1 silent fusion gene in residual dormant leukemia cells has been implicated as a possible cause of late relapse in ALL due to acquired changes that take place over time [9]. These changes have not been demonstrated in patients with LBL; however gene expression profiling has been used to distinguish whether certain subtypes of T-LBL are more favorable than others [10, 11]. The rate of bcl-2 expression seems to be linked to drug sensitivity. Patients with higher expression of bcl-2 have been identified to have TAL1 and LYL1 and demonstrate a drug resistance pattern while those expressing HOX11 have lower bcl-2 levels and a more favorable prognosis [10, 11].

The success of the initial treatment and the duration of the first clinical remission are very important predictors of prognosis [8]. BM involvement at initial diagnosis has a higher propensity for relapse. Survival for early BM relapse has been reported to range from 0 to15% [12], whereas those with late BM relapse range from 14 to 50% [13]. In addition, the type and intensity of the chemotherapeutic regimen given may also contribute to the overall outcomes of these patients. In earlier studies, the use of standard lymphoma regimens such as CHOP in patients with LBL yielded complete response (CR) rates ranging from 40 to 70% [14]. More recently, intensive pediatric ALL regimens have been utilized in patients with adult ALL and LBL. In earlier studies, the use of standard lymphoma regimens such as CHOP in patients with LBL yielded complete response (CR) rates ranging from 40 to 70% [14]. More recently, intensive pediatric ALL regimens have been utilized in patients with adult ALL and LBL. Thomas et al. describes the use of HyperCVAD in adult patients with predominant T-LBL [2]. CR was achieved in 91% of patients using this protocol. The 3-year progression free survival (PFS) and overall survival (OS) of 33 patients with LBL treated with HyperCVAD had been 66% and 70%, respectively. The 3-year PFS and OS for patients with a T-cell phenotype in particular were 62% and 67%, respectively. Because of these encouraging results we elected to use HyperCVAD in our patient.

L-asparaginase has been described to be an integral part of many pediatric regimens [15, 16]. Its antileukemic effect is thought to result from the reduction of circulating asparagine. GMALL 04/89 and GMALL 05/93 protocols [5] were modeled after pediatric ALL regimens and include asparaginase during the induction phase. Hoelzer et al. describes the use of these regimens in 45 adult patients with T-LBL [5]. Overall, 42 out of 45 patients (93%) achieved a CR. Estimated OS, continuous CR, and DFS at 7 years were 51%, 65%, and 62%, respectively. We incorporated L-asparaginase into the HyperCVAD regimen because of its fundamental use in pediatric ALL protocols and previous success in treatment of T-LBL [5].

Autologous stem cell transplant remains the standard approach for consolidation in first or second remission. This was offered to the patient yet she declined.

To our knowledge, this case is the latest reported relapse of T-LBL at 16 years after the first clinical remission. The patient was successfully treated at relapse with HyperCVAD and L-asparaginase and now remains in second remission 5 years from first relapse.
